# The Development of an mHealth Tool for Children With Long-term Illness to Enable Person-Centered Communication: User-Centered Design Approach

**DOI:** 10.2196/30364

**Published:** 2022-03-08

**Authors:** Angelica Wiljén, John Eric Chaplin, Vanessa Crine, William Jobe, Ensa Johnson, Katarina Karlsson, Tomas Lindroth, Anneli Schwarz, Margaretha Stenmarker, Gunilla Thunberg, Joakim Öhlén, Stefan Nilsson

**Affiliations:** 1 Institute of Health and Care Sciences Sahlgrenska Academy University of Gothenburg Gothenburg Sweden; 2 University of Gothenburg Centre for Person-Centred Care Sahlgrenska Academy University of Gothenburg Gothenburg Sweden; 3 Department of Paediatrics Region Västra Götaland, Södra Älvsborg Hospital Borås Sweden; 4 Department of Paediatrics, Institute for Clinical Sciences Sahlgrenska Academy University of Gothenburg Gothenburg Sweden; 5 Department of Informatics School of Business, Economics and IT University West Trollhättan Sweden; 6 Centre for Augmentative and Alternative Communication University of Pretoria Pretoria South Africa; 7 Department of Inclusive Education University of South Africa Pretoria South Africa; 8 Department of Health Sciences, Faculty of Caring Science, Work Life and Social Welfare University of Borås Borås Sweden; 9 Department of Applied Information Technology University of Gothenburg Gothenburg Sweden; 10 Department of Research, Education and Innovation Region Västra Götaland, Södra Älvsborg Hospital Borås Sweden; 11 Department of Paediatrics Region Jönköping County Jönköping Sweden; 12 Department of Clinical and Experimental Medicine Linköping University Linköping Sweden; 13 Dart Centre for Augmentative and Alternative Communication and Assistive Technology Sahlgrenska University Hospital Gothenburg Sweden; 14 Speech and Language Pathology Unit, Institute of Neuroscience and Physiology Sahlgrenska Academy University of Gothenburg Gothenburg Sweden; 15 Palliative Centre Sahlgrenska University Hospital Region Västra Götaland Gothenburg Sweden

**Keywords:** children, communication, long-term illness, mHealth, pediatric care, person-centered care, symptom assessment, universal design

## Abstract

**Background:**

Children with long-term illnesses frequently experience symptoms that could negatively affect their daily lives. These symptoms are often underreported in health care. Despite a large number of mobile health (mHealth) tools, few are based on a theoretical framework or supported by scientific knowledge. Incorporating universal design when developing a product can promote accessibility and facilitate person-centered communication.

**Objective:**

The aim of this study is to identify the symptom-reporting needs of children with cancer and congenital heart defects that could be satisfied by using a mobile app. Another aim is to evaluate how the child might interact with the app by considering universal design principles and to identify parents’ views and health care professionals’ expectations and requirements for an mHealth tool.

**Methods:**

User-centered design is an iterative process that focuses on an understanding of the users. The adapted user-centered design process includes 2 phases with 4 stages. Phase 1 involved interviews with 7 children with long-term illnesses, 8 parents, and 19 health care professionals to determine their needs and wishes for support; a workshop with 19 researchers to deepen our understanding of the needs; and a workshop with developers to establish a preliminary tool to further investigate needs and behaviors. Phase 2 involved interviews with 10 children with long-term illnesses, 9 parents, and 21 health care professionals to evaluate the mock-up (prototype) of the mHealth tool. Data were synthesized using the interpretive description technique.

**Results:**

A total of 4 aspects of needs emerged from the synthesis of the data, as follows: different perspectives on provided and perceived support; the need for an easy-to-use, non–clinic-based tool to self-report symptoms and to facilitate communication; the need for safety by being in control and reaching the child’s voice; and a way of mapping the illness journey to facilitate recall and improve diagnostics. The children with long-term illnesses expressed a need to not only communicate about pain but also communicate about anxiety, fatigue, fear, and nausea.

**Conclusions:**

The findings of this study indicated that the PicPecc (Pictorial Support in Person-Centered Care for Children) app is a potential solution for providing communicative support to children with long-term illnesses dealing with multiple symptoms and conditions. The interview data also highlighted symptoms that are at risk of being overlooked if they are not included in the mobile app. Further studies are needed to include usability testing and evaluation in hospitals and home care settings.

## Introduction

### Background

Children with long-term illnesses such as cancer [1] and congenital heart defects [2] have multiple symptoms that cause discomfort and negatively affect their daily lives. These children have a significantly lower health-related quality of life (HrQoL) than that of healthy controls [3,4]. Children experiencing multiple symptoms also have lower HrQoL than that of children with fewer symptoms [5]. Parents of children with long-term illnesses, such as cancer, confirmed that they experience challenges in identifying symptoms—specifically pain-related symptoms—in their children [6,7]. As a result, these symptoms are often underreported and not identified [8,9], treated, or relieved [10], causing children unnecessary distress and pain.

Symptom assessment is a key to symptom relief, and children should have the opportunity to self-assess and report their symptoms. However, such reporting may be limited by the children’s ability to measure and describe distinct cancer-related symptoms, which can further affect the ability of parents or health care professionals to provide the necessary supportive care [11]. Evidence to show that exclusive use of rating scales makes a difference in the management of symptoms, such as pediatric pain, is limited [12]. More knowledge is needed about how symptom assessment could be combined with other measures to reduce symptom intensity and improve function [12]. There is also a need to ensure valuable apps continue to be used by children and are usable by providing access to maintenance and updates [13,14]. Therefore, finding an appropriate means of facilitating children’s self-reported communication of symptoms is important. Bernier Carney et al [15] and Wesley and Fizur [16] proposed the use of mobile health (mHealth) tools such as mobile gamified apps that include creative approaches to symptom assessment to enable health care professionals and parents to capture self-reported data of children’s symptoms.

In existing apps, pain is often considered as a core symptom to be monitored via self-report [15]. Apps can even help to significantly reduce cancer-related pain scores, especially when they offer instant messaging modules [17]. Besides pain, other symptoms such as changes in appetite, cough, dizziness, nausea, fatigue, difficulty sleeping, vomiting, and well-being are also represented in apps [18-20]. In general, the willingness to use these apps is high because of developmentally appropriate interfaces and features that ensure child-centered self-reporting [17-21]. No specific symptom management app was found for children with congenital heart defects.

When developing an mHealth supportive tool or app for children, it is important to consider children’s diagnoses and their cognitive, developmental, and language levels [22,23]. It is also necessary to take into account children’s experiences and suggestions. However, children are rarely invited to participate in the development of such tools [24]. According to a qualitative evaluation of existing apps for pain management (n=36), it was stated that most apps were developed without end user and clinician involvement. In addition, the apps had security problems, lacked graphical data visualization, and did not include instruments used in clinical settings [25]. Moreover, measures are generally created for adults and simplified at a later stage to accommodate use by children under the assumption that children and adults share the same concerns [26].

To ensure that children’s preferences and needs are incorporated in the mHealth tool, it is important to include children, as well as other stakeholders, in its development. This process is referred to as user-centered design (UCD) and involves all stakeholders working together as equal partners to contribute to the design of a new product (eg, mHealth tool) in an attempt to develop an efficient and feasible tool for a specific population [27,28]. When designing an app that can be used by various people in different situations, it is important to take human abilities, needs, and requirements into account. As such, universal design (UD) or *inclusive design* aims to facilitate accessibility for users with all kinds of abilities and needs [29]. Accessibility could be provided through the use of pictures, audio, and easy-to-read texts [30-32].

Three different approaches to centeredness exist in pediatric health care: child, family, or person [33]. This study focuses on person-centeredness, as this closely follows the caring process and includes the child and the family [34]. In pediatric symptom assessment, a person-centered approach could help in selecting and providing optimal treatments [26,35]. In health care, the need to implement person-centered care is growing [36,37]. Person-centered care aims to create partnerships among patients, families, carers, and health care professionals [34,37]. As defined by the Gothenburg model for person-centered care, the three routines of this approach are as follows: (1) initiating a partnership by eliciting the patient’s narrative, including goals, capabilities, and limitations; (2) building the partnership through the cocreation of a health plan promoting the patient’s self-efficacy and self-care; and (3) safeguarding the partnership by documenting the patient’s story and health plan to support the continuity of care [37,38]. A European Union standard for patient involvement has recently been launched to facilitate pediatric person-centered care [34,39].

### Objective

This study is based on the child’s perspective, meaning that the child’s needs and experiences are central. It strives for a child-centered approach by listening to the child’s preferences and taking into account the adults’ views on what is in the best interests of the child [34,39,40]. Research shows that parent-centered communication styles can feel disempowering for children, whereas communication tailored to child-centric communicative and developmental needs gives them a sense of respect, safety, and control. In turn, empowering children and promoting their autonomy and partnership may be beneficial for their quality of care, health outcomes, and well-being [1].

First, our study aims to identify the symptom-reporting needs of children with cancer and congenital heart defects, with and without communication challenges, to inform the design of a tool that could be used in a mobile app to meet the child’s requirements in order for them to feel safe in its use to communicate their symptoms. As most apps are focused on pain and not necessarily on other symptoms such as anxiety, nausea, and fear, this study aims to describe the initial development of a person-centered communication support mHealth tool (ie, PicPecc [Pictorial Support in Person-Centered Care for Children]) intended for use as a self-report device by children with long-term illnesses to report and manage their symptoms at a hospital or at home. Children with cancer and children with congenital heart defects were chosen as they often experience multiple symptoms, which would test the functionality of the app. The subsequently developed app is called PicPecc.

Second, this study aims to evaluate how the child might interact with the app, considering UD principles, and to identify parents’ views and health care professionals’ expectations and requirements for an mHealth tool.

## Methods

### The Adapted UCD Process

UCD is an iterative process that focuses on an understanding of the users and their context in all stages of design and development. In UCD, the design project is based on an explicit understanding of the users, tasks, and environments. Therefore, the team involved in the design process should include a range of professionals across multiple disciplines, as well as domain experts, stakeholders, and the users themselves [41,42]. This is a way of increasing the impact of mHealth tool usability for children and adolescents [27,28]. During the development process of PicPecc, a set of key principles were in focus: dynamism, iteration, creativity, openness to change, and a look forward toward future evaluation and implementation [43].

From a user-centered perspective, it is central to not only involve the user but also to have a point of departure in the existing practice where the artifact is intended to be used [44]. Thus, being user-centered means having a focus on multiple users and on the context in which the use takes place. Thus, in a care situation involving children, it is central to weave in the experiences of children, their parents, and health care professionals [41]. In addition to users, UCD stresses the importance of having different types of experts in the design process. We are not referring primarily to information technology experts but to experts in the areas that the design process may affect.

From a UCD perspective, design is not just the activities conducted together with a user or activities for which the digital artifact is developed. A central part of the process is to set the framework for the project and define its starting point. It is said that design is vision driven and intentional, meaning it is consciously aimed toward change [45]. Thus, the design process starts as soon as the principles for the project are defined and data are collected and lasts beyond the implementation of the artifact in the practice it was designed for.

In this study, the starting point was a design approach that departs from 3 child-centered standpoints. The first is to design a solution that solves the problem of children’s symptoms being underreported and not identified [8,9], treated, or relieved [10], causing children to experience unnecessary distress. The second is symptom assessment being key to symptom relief, whereby children should have the opportunity to self-assess and report their symptoms. Thus, this study centers on children’s ability to self-report their symptoms. The third standpoint was to follow the principles of UD to identify the expectations and requirements of the health care professionals who would analyze the data and the parents’ attitude toward using the app and, finally, to use a mock-up of the app to evaluate how children from different backgrounds, speaking different languages, and with different long-term illnesses benefit from the app.

**Figure 1:**
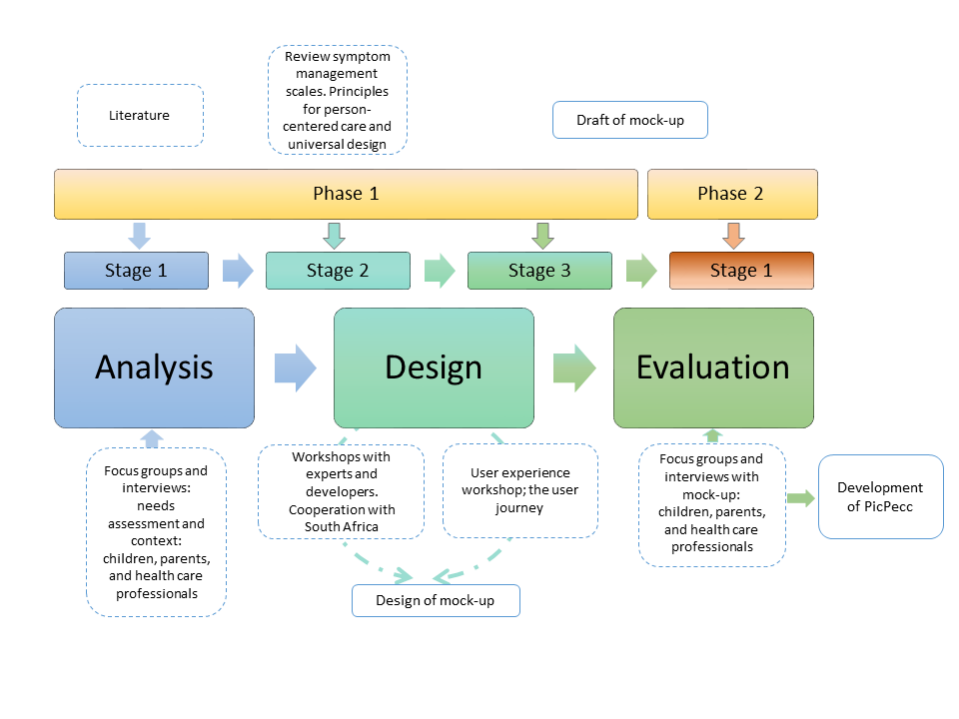
A visual presentation of the two phases of the study. PicPecc: Pictorial Support in Person-Centered Care for Children.

Inspired by previous research, the UCD process included feedback, suggestions, and observations from a multidisciplinary research team, children, parents, and health care professionals in 2 phases [46]. Figure 1 shows a visual presentation of the 2 phases.

Children with cancer could not participate in all stages of UCD for medical and ethical reasons. An adapted version of UCD was used where the children’s, parents’, and health care professionals’ voices were represented in stages 2 and 3 via interview data. Subsequently, the adapted UCD was followed in the development of the PicPecc mHealth tool, comprising 2 sequential phases.

Phase 1 involved 3 stages. For example, in stage 1 of phase 1, interviews with children with long-term illnesses and their parents were conducted, with participants sharing their lived experiences to guide the development of the PicPecc app. Health care professionals who worked within the system also provided input on their needs and the perceived needs of the children when caring for them. The children’s needs and wishes were the departure points for all the following design activities, followed by the results from the parents and health care professionals.

Stage 2 of phase 1 involved a workshop where researchers with theoretical and clinical intervention knowledge presented information on person-centered care, UD, and evidence-based practices on the use of mHealth tools in various settings, such as high-, low-, and middle-income countries, to inform the development needs of the specific intervention (PicPecc).

In stage 3 of phase 1, a workshop was held with the design team (information technology personnel). These designers provided valuable input in terms of which electronic platforms might best suit the needs of the specific tool (PicPecc). In addition, this stage involved using the analyzed data from the first 2 stages as guidance for the development of PicPecc. The task of the experts and developers was to highlight existing evidence and indicate what technical possibilities were available to meet the children’s requirements. The design of the app was decided along with experts in UD [47] to facilitate accessibility for all users, regardless of their ability and needs.

In phase 2, we followed principles from previous research and included a pilot test with children, parents, and health care professionals who tested our ideas for the first time with a mock-up (prototype) of the developed PicPecc [48]. Stage 1 (forming part of phase 2) involved interviews with children, parents, and health care professionals to evaluate the PicPecc app (Figure 1). The participants tested the ideas and reflected on the flow of the PicPecc app, commenting on whether the app was easy or fun to use. The participants also described whether the app was adaptable to their individual needs.

The Shier [49] Pathway to Participation model (2001) is often used to assess commitment to youth participation. The model contains 5 levels of participation, where level 5 means that children share power and responsibility in the development of an intervention. The present adapted UCD reaches level 3 on the Shier [49] model; that is, children’s voices are taken into account in the process of developing the intervention.

### Ethical Approval

After ethical approval was obtained from the Swedish Ethical Review Authority (reference 2019-02392; 2020-02601; 2020-06226), children and parents were contacted and informed about the study through assigned persons working in the pediatric wards where the children were treated. Each assigned person was informed about the study by the researchers.

### Phase 1

#### Stage 1 Semistructured Interviews and a Focus Group

##### Research Question

How do children with long-term illnesses, their parents, and health care professionals prefer to communicate about symptoms and symptom relief, and what symptoms are important to talk about?

##### Participants

Participants were recruited from 5 different hospitals in Sweden. Purposive sampling of children, parents, and health care professionals was used according to the following eligibility criteria: (1) an understanding of Swedish or English, (2) an age of 5 to 17 years, (3) a cancer or congenital heart defects diagnosis, and (4) an experience of illness. We chose to include children with an expected experience of a range of symptoms. Children in need of end-of-life care were excluded. Parents of children diagnosed with cancer or congenital heart defects, as well as health care professionals working with children with these diagnoses, were also involved in this study. Health care professionals with <6 months of work experience in pediatrics were excluded. Participants in this first stage of phase 1 included 7 children with cancer, 8 parents, and 19 health care professionals (Table 1).

**Table 1:** Characteristics of participants in phase 1 (stage 1) and phase 2 (stage 1).

Characteristics of health care professionals				Phase 1 (stage 1), n/N (%)		Phase 2 (stage 1), n/N (%)		Participants who participated in both phases, n/N (%)
**Health care professionals**								
	**Gender**							
		Female	15/19 (79)		18/21 (86)		9/11 (82)	
		Male	4/19 (21)		3/21 (14)		2/11 (18)	
	Registered nurse		11/19 (58)		17/21 (81)		7/11 (64)	
	Assistant nurse		3/19 (16)		1/21 (5)		1/11 (9)	
	Physician		5/19 (26)		3/21 (14)		3/11 (27)	
	**Working years in the same workplace**							
		0-5	4/19 (21)		9/21 (43)		3/11 (27)	
		6-15	6/19 (32)		2/21 (10)		1/11 (9)	
		≥16	9/19 (47)		10/21 (48)		7/11 (64)	
**Children and parents**								
	Children		7/15 (47)		10/19 (53)		6/11 (55)	
	Parents		8/15 (53)		9/19 (47)		5/11 (45)	
**Age (years) of the child during the time of data collection**								
	7-11		2/7 (29)		4/10 (40)		2/6 (33)	
	12-14		4/7 (57)		5/10 (50)		3/6 (50)	
	≥15		1/7 (14)		1/10 (10)		1/6 (17)	
**Diagnosis of the child at the time of data collection**								
	Leukemia (ALL^a^ or AML^b^)		5/7 (71)		6/10 (60)		5/6 (83)	
	Solid tumor		2/7 (29)		2/10 (20)		1/6 (17)	
	Congenital heart defects		0/7 (0)		2/10 (20)		0/6 (0)	
**Time since diagnosis at the time of data collection (months)**								
	0-3		2/7 (29)		0/10 (0)		0/6 (0)	
	4-8		4/7 (57)		1/10 (10)		0/6 (0)	
	≥9		1/7 (14)		9/10 (90)		6/6 (100)	

^a^ALL: acute lymphoblastic leukemia.

^b^AML: acute myeloid leukemia.

##### Procedure

The health care professionals were contacted by the last author (SN) and invited to participate. Interviews were conducted with children and parents at the hospital where the child had a scheduled appointment for treatment. Written consent was obtained from health care professionals and parents, and assent was obtained from each child. The interviews were conducted by the first (AW) and last authors (SN) and were audio recorded or video recorded (Textbox 1). The young children were given the option to be interviewed together with their parents or to be interviewed alone. To hear the child’s own voice, the child was asked to answer the question first, after which the parent could answer the question to add depth to what the child said and to add the parents’ view. Some health care professionals also chose to do their interviews in dyads, as they often worked together. Upon receiving consent, a suitable time and a meeting place were decided. The health care professionals’ interviews were conducted at their workplace. A focus group with 4 health care professionals was also conducted, where the first author (AW) was the moderator and the last author (SN) was an observer. At the end of each interview, all participants were asked to prioritize the different symptoms according to their importance on a 5-point Likert scale (Table 2). The symptoms were identified as important, in accordance with previous literature [50].

**Topics and questions of the semistructured interviews.**

**Data collection**

- Stage 1 (phase 1)

**Topics**

- Existing support, existing scales, and experiences of symptoms and conditions
- A communicative support tool as support when dealing with multiple symptoms and conditions

**Questions**

- Tell me about a good and a bad care situation?
- What symptoms have you experienced (children), and which symptoms do you see (adults)?
- How do you measure what the child feels (adults)?
- What kind of support is there when dealing with multiple symptoms and conditions?

**Table 2:** Prioritization of symptoms that need to be assessed in a new way (N=27).

Symptoms	Strongly disagree (1), n (%)	Disagree (2), n (%)	Neutral (3), n (%)	Agree (4), n (%)	Strongly agree (5), n (%)	Values, mean (SD)
Anxiety	0 (0)	0 (0)	1 (4)	10 (37)	16 (59)	4.56 (0.57)
Fatigue	0 (0)	3 (11)	8 (30)	11 (41)	5 (19)	3.67 (0.90)
Fear	0 (0)	0 (0)	1 (4)	14 (52)	12 (44)	4.41 (0.56)
Nausea	1 (4)	5 (19)	5 (19)	6 (22)	10 (37)	3.70 (1.24)
Pain	2 (7)	5 (19)	6 (22)	8 (30)	6 (22)	3.41 (1.23)
Well-being	0 (0)	1 (4)	4 (15)	16 (59)	6 (22)	4 (0.72)

Multi-perspective data with children, parents, and health care professionals were collected through semistructured interviews—one-on-one children–parent, parent-parent, and health care professional dyads and in a focus group. Multiple data collection approaches were a viable option to ensure different perspectives [51]. Multi-perspective data also enabled us to compare different participants’ perspectives and feelings regarding symptom relief and explore the possible underlying reasons for differences.

The individual interviews with the children (4/7, 57%) lasted between 17 and 44 minutes (mean 35, SD 10.56 minutes) and interviews with the parents (5/8, 63%) lasted between 26 and 37 minutes (mean 30.40, SD 5.39 minutes), one of the interviews were a dyad interview with a mom and a dad. The dyad interviews with the child and parent (3/12, 25%) lasted between 29 and 60 minutes (mean 45.33, SD 12.71 minutes). The individual interviews with health care professionals (11/19, 58%) varied in length from 28 to 53 minutes (mean 38.36, SD 8.16 minutes). The health care professionals’ dyad interviews were 53 minutes and 58 minutes long (mean 55.50, SD 2.5 minutes), respectively. A focus group was conducted with 21% (4/19) of the health care professionals to generate more variation and multiple perspectives in the collected data. The length of the focus group interview was 66 minutes.

##### Data Analysis: Qualitative Data

All audio data were transcribed verbatim and complemented with video recorded information. The qualitative analysis software NVivo 12 Pro (QSR International) was used to arrange and rearrange the codes into patterns and relationships.

The analysis process started with the transcribed interviews being read intensely by the first (AW) and third (VC) authors to obtain a sense of the whole, in accordance with interpretive description [51]. During the preliminary reading, words and sentences that corresponded to the aim of the analysis were underlined according to a broad inductive coding. The different groups of participants were first coded sequentially, starting with the children’s interviews, followed by the parents, and finally, the health care professionals. The initial coding was used as a basis for subsequent coding; however, new codes were added for parents’ and health care professionals’ interview data. Tentative patterns and relationships were identified. A broader analysis was subsequently made where questions such as, “What does it mean?” and “What are they talking about?” were asked. Continued synthesis of the tentative patterns facilitated the understanding of various interpretations of the topic. The data reflected not only common patterns but also ideas and beliefs. The final step of the analysis was the definition of themes [52].

During the analytical process, the researchers were mindful that their preunderstanding could affect the data and, therefore, returned to the data repeatedly for confirmation of the emerging patterns and relationships. These patterns and relationships were discussed within the research team to reach a consensus about relevant findings and to ask, “What are we not seeing?” The analytical process from the first phase gave us an understanding of the unmet needs and wishes mentioned by participants when dealing with multiple symptoms.

Member checking was conducted with the participants as an important step to improve qualitative validity [51].

Interpretive description supports a credible and transparent process in qualitative research [52,53]. The researchers need to make their preconceptions regarding the research topic transparent; that is, recognize that these preconceptions can influence data collection or data analysis. This was performed in an interdisciplinary research group with members who had several years of experience in interaction design, information systems, nursing sciences, pediatric oncology care, psychology, communication, and UD. We strove to be as open as possible to the participants’ perspectives and experiences.

##### Data Analysis: Quantitative Data

All assessments from the participants were described using descriptive statistics; that is, with numbers, percentages, and means.

#### Stage 2 Workshop

The project team arranged a workshop to ensure theoretical relevance and anchoring regarding relevant clinical, person-centered, and design-related perspectives.

##### Research Question

Which theoretical knowledge and findings from the research are relevant to consider in the development and research of the app?

The workshop was designed explicitly to prevent such shortcomings from occurring in this project. To ensure that our app of UD principles was acceptable in a different societal context, a collaboration was established with university partners in South Africa. We wanted to investigate whether our app based on the principles of UD was acceptable in a completely different context. This context also includes a multilingual and multicultural environment that poses additional challenges.

##### Participants

The workshop was attended by 19 researchers, 12 health care professionals, and 8 postgraduate students from Sweden and South Africa in areas related to the project.

##### Procedures

Topics of discussion involved person-centered care, communication of children in health care settings, principles of UD and communication support, and the design and development of mHealth tools.

The workshop was video recorded, and notes were taken; mapping of relevant theoretical perspectives and patterns were conducted.

##### Data Analysis

The data collected from the workshop were analyzed using manifest analysis [54]. In the analysis process, important areas were selected to be used in stage 3. This workshop provided knowledge of the conditions for using an app in low- and middle-income countries. The workshop also mapped existing apps and in-depth knowledge of person-centered care and UD.

#### Stage 3 Workshop

We determined the possible user journey for the PicPecc app by means of a user experience (UX) workshop.

##### Research Question

What is a possible user journey for the PicPecc app?

##### Participants

Throughout the project, the children were included in the research and design whenever possible. However, these children had decreased immune function and experienced fatigue. This made it difficult to include them in design events such as workshops. However, other users of the mHealth tool (eg, nurses) participated in the design workshop. Web-based participation was considered and can be of value [55,56]. However, some activities were not suited for web-based settings, and web-based participatory design settings have proved to be challenging [57].

A total of 3 UX specialists, 2 speech and language therapists (a PhD and an associate professor), 3 pediatric nurses (a PhD student, a PhD, and an associate professor), 2 researchers in information systems (both PhDs), and a health psychologist specialist (an associate professor) participated in stage 3.

##### Procedures

A full day, face-to-face interprofessional workshop was held in Gothenburg, Sweden, to collate the assumptions of the different researchers into a design report. The workshop had a structured agenda comprising 3 distinct parts. The first part comprised a value proposition canvas with the purpose of pinpointing the value offering [58]. The second part focused on impact mapping with the purpose of tracing how and who created the impact [59]. Finally, a user journey was undertaken to create a visualization of a user’s possible interaction with the app over time [60].

##### Data Analysis

This workshop provided knowledge for the developers and gave them a basis for developing a mock-up.

### Phase 2—Stage 1 Semistructured Interviews and Focus Groups

Phase 2 involved only 1 stage where children with cancer or congenital heart defects, their parents, and health care professionals provided input on the mock-up version of the PicPecc app.

#### Research Question

How can the PicPecc app provide children, parents, and health care professionals support when communicating about symptoms and symptom relief?

#### Participants

Participants were recruited from 5 different hospitals in Sweden. The recruitment process was similar to stage 1 of phase 1. These participants were also invited to participate in phase 2, resulting in 6 children, 5 parents, and 11 health care professionals (Table 1) taking part in both phases. Additional recruitment of participants was conducted. Finally, the sample comprised 10 children with cancer or congenital heart defects, 9 parents, and 21 health care professionals (Table 1).

#### Procedure

The procedure was similar to stage 1 (phase 1), apart from using the mock-up as stimuli material (Textbox 2).

**Topics and questions of the semistructured interviews.**

**Data collection**

- Stage 1 (phase 2)

**Topics**

- Process of engaging with the communicative support tool
- Existing websites for support and tips when dealing with symptoms and conditions

**Questions**

- What do you want to know from the support tool?
- What do you want help with?
- What motivations do you and the children need to use it?
- When can it be useful?
- Did you understand everything?

The individual interviews with the children (7/10, 70%) varied from 18 to 57 minutes (mean 37.29, SD 11.80 minutes), the individual interviews with the parents (6/9, 67%) varied from 19 to 62 minutes (mean 37.67, SD 13.77 minutes), and the dyad interviews with a child and a parent (3/16, 19%) were between 23 and 41 minutes (mean 32.33, SD 7.36 minutes). The individual interviews with the health care professionals (11/21, 52%) varied from 26 to 71 minutes (mean 44.82, SD 10.60 minutes) and the 2 focus group interviews with health care professionals (10/21, 48%) lasted between 37 and 75 minutes (mean 56, SD 19 minutes). In the 2 focus groups, 30% (3/10) of participants took part in the first focus group, and 70% (7/10) of participants took part in the second focus group.

#### Data Analysis: Qualitative Data

In stage 1 of phase 2, all participants were presented with the prototype of the mHealth tool that was developed according to the key findings of the first phase.

We followed the same data analysis procedures as in stage 1 of phase 1.

## Results

### Phase 1

#### Stage 1—Qualitative Findings

The results are presented with quotations, which are presented with a unique code and general information about the speaker.

##### Significant Standpoints to Address

Children and parents emphasized a need to address both the positive and negative sides of daily life. Health care professionals focused to a higher degree on symptoms in need of treatment and requested valid instruments. Participants stressed highlighting symptoms beyond pain; for example, anxiety or fear.

All participants talked about the ways in which symptoms interrupt the child’s life. Self-care strategies are needed to help children cope:

Not meeting friends and not even doing...video games make them feel sick. That’s it. And some of them feel ill as long as the therapy lasts.

310; female pediatrician

##### Different Perspectives on Provided and Perceived Support

Children expressed a wish to receive support for self-care; however, there seems to be a disparity between what children want and what health care professionals provide. Parents experienced a lack of support from health care professionals who worked closely with the child. Health care professionals focused more on physiological symptoms, whereas children and parents indicated that they also needed psychosocial support. Children and parents also appreciated when the nurses provided emotional support and took time and stayed a little longer to talk about entertaining topics such as movies or books:

For me it was most useful and valuable when the nurses stayed for a bit and just talked. I saw that some of the nurses had an easy way of talking with my daughter about movies and...they were more open for conversations.

202; mother of a 14-year-old girl

##### Need for an Easy Tool to Assess Symptoms and Facilitate Communication

Children, parents, and health care professionals expressed a need for an easy way of assessing the child’s symptoms in a reliable manner based on multimodal strategies. For example, health care professionals were satisfied with existing pain scales but at the same time stated that these scales were not used as often as they could be. Health care professionals needed an easy way of assessing pain or other symptoms, as they sometimes doubted the assessment given by the child, based on the signs and behavior they could observe. Parents wished that they could facilitate the children’s expression of how tired or how much pain they experienced in different ways, such as via visual support. The children wanted to use an easy tool that helped them explain how they felt, sometimes even without having to talk because of feeling tired or experiencing pain:

It would be easier, instead of talking all the time, you could just show them.

104; boy, 16 years

If you have a bad day, then you may not want someone to ask how you feel. Then you can write it in the app. That would be a smart thing.

102; girl, 14 years

##### Creating Safety and Reaching the Child’s Voice

All participants stressed the importance of safety in the situation. From the child’s perspective, this was expressed as a need to feel that they have control over the situation. Parents and health care professionals described wanting to access the child’s needs and wishes; that is, get to the primary source. Children wanted to feel safe and have a sense of control over the situation. When they knew what to expect, children could handle their treatment-related procedures better. Health care professionals felt they were able to listen to the child’s needs and wishes and, thereby, provide appropriate help:

I believe in the visual for a child...That the child has a way to show and express, so that you don’t lean too much on the parent’s interpretation.

309; female pediatrician

##### Mapping the Journey to Facilitate Recall

The ability to visualize and thereby easily remember the child’s journey was emphasized. Children, parents, and health care professionals wanted to measure the aspects of well-being and not only the negative experiences, as described by the following adolescent:

It would have been good to have a positive thing, so that you can see that sometimes you feel well, so you can see which days during the week are the better ones.

104; boy, 16 years

Another perspective mentioned by health care professionals was the need to retrieve information with the purpose of providing appropriate symptom relief to the individual child.

Parents stressed the need to strengthen their child’s self-efficacy using an mHealth tool to prompt and support the child in symptom relief.

##### Quantitative Results

All symptoms were found to be important, as shown in Table 2.

#### Stage 2

The presentations and the following discussion created a common ground regarding four areas. (1) The discussion resulted in a mutual understanding of the most relevant symptoms (anxiety, fatigue, fear, nausea, and pain), the assessment of symptoms (Visual Analog Scale, Numerical Rating Scale, Faces Pain Scale–revised, and Wong–Baker Faces Pain Rating Scale), and management of symptoms. Regarding the assessment of symptoms, for instance, the project identified a need for digital assessment of pain that aligned with the current assessment method at different hospitals. (2) There was also a focus on children’s rights, person-centered care, and UD from an augmentative and alternative communication perspective. This discussion emphasized the development of an mHealth tool with communication support and illustrations of specific actions and text-augmented communication for children of different ages, cultures, and cognitive capabilities. The idea of a UD perspective focused on alternative communication became a central thesis for this project. (3) Another outcome was a deeper understanding of UCD, where the focus is on the use situation and how the mHealth tool also must fit into a larger context of information systems to stay relevant beyond the study phase. (4) Finally, there was a slightly different discussion on how to measure the effect of the intervention from a neurochemistry perspective; that is, the possibility to measure the effects of the intervention in blood samples.

A meta outcome of the second phase acknowledged the complexity of the intervention at hand. Designing a research-anchored mHealth tool from a UD perspective is a great challenge in itself. Designing something that fits into the existing care practices and information systems at different locations increased the complexity even further.

#### Stage 3

Although the children did not participate in this stage, their perspectives were central throughout the workshop. From stage 1, the children’s needs and wishes were fundamental to each part of this study.

The value proposition canvas summarized the different stakeholders and their pains, gains, and actions. For instance, from the child’s perspective, a typical symptom would be fear in relation to the disease. A typical gain would be a visualization of their symptom journey, and a typical action would be to assess their well-being. This was followed by detailing the possible impacts of the app.

The outcome from the workshop was translated into a number of user stories (participant journeys); that is, a set of requirements based on different actions that different users want to perform with the system to fulfill certain goals. These user stories were grouped into themes. These themes could be general; for example, *user management* and *calendar*. More specific ones were *gamification*, *my pets*, *avatar system,* and an *assessment system*. *My pets* and the *avatar system* are related to the personalization of the system. *Gamification* is related to motivating the child to perform the assessments (Multimedia Appendix 1).

### Phase 2

#### Stage 1

In stage 1 (also referred to as phase 2), children, their parents, and health care professionals provided input on the mock-up version of the PicPecc app.

##### Different Perspectives on Provided and Perceived Support

Health care professionals in this phase also focused more on physiological symptoms, whereas children and parents indicated that they also needed psychosocial support (Multimedia Appendix 2). The children talked about how the PicPecc app could be a way of communicating without having to speak to either their parents or health care professionals. Children also said that this could help them express how they feel and help them manage their symptoms:

It helps you to say how you feel, and the tips on what you can do to lessen the pain or lessen the nausea; it’s good to get help with that because sometimes it feels like nothing works. But if you have tips, maybe you will find one that works for you.

101; girl, 14 years

Health care professionals also emphasized the child’s self-care and further wanted the PicPecc app to provide professional support, such as the next dose of medication, preparations for procedures, and information about possible side effects.

The health care professionals thought they could see the PicPecc app being useful during medical rounds to follow up on symptoms to guide treatment and prepare the child for treatment and procedures. This was facilitated by a function in the app; that is, diagrams.

##### Need for an Easy Tool to Assess Symptoms and Facilitate Communication

Children thought that gamification (eg, in PicPecc, the use of the app enabled collection of pets) could motivate the use of the app. Health care professionals were also of the opinion that a reward system was a positive motivator for children to use the app. Older children stated that the app was an easy way of informing health care professionals and parents about how they felt. The children also felt that their parents might ask fewer questions if they (the parents) could have access to the information on how their child assessed his or her symptoms in PicPecc:

This app could make mum and dad stop asking how I feel all the time; instead I can go in here and press from time to time how I feel, so they can see.

101; girl, 14 years

Health care professionals and older children thought the calendar or schedule was also a motivator as it would help the children during hospital visits. This was also verified by a parent:

I’ve been giving my child medicine now at two o’clock and the next one comes at eight o’clock. And she wanted to know that because then she knew she’ll feel better then.

201; mother of a 14-year-old girl

The health care professionals mentioned that PicPecc might clarify their communication with the children and especially envisioned using it with children who found expressing their opinions challenging. The PicPecc app can also facilitate understanding of the child:

It will probably be easier to ask the child than...Now you ask the parents, even if the child can talk. So, you don’t reach the child, it’s the parents. Here I think I can reach them.

316; focus group, nurse in pediatric cardiology

The colors and faces added to the thermometer in PicPecc helped with the understanding of the thermometer scale. Most symptoms were easy to assess, ranging from feeling good (green and smiley face) to worse (red, sad, and crying face), except for the symbol *appetite*, where the participants found this kind of scale difficult to use.

The health care professionals liked that the mock-up included a page called *my page*, where the child could write their requests and wishes for their care. However, some parents doubted that their child would use that function. Nevertheless, the health care professionals saw it as a means of helping the child to become more involved in their own care.

Parents appreciated the diagram function and the possibility for the child to assess symptoms using the body figure. The feedback provided by PicPecc could help the child to realize that they need help with symptom management. The feedback option may also offer a way for the parent to help explain feelings that the child might find difficult to express clearly.

The word *anxiety* was not problematic for the children. However, health care professionals and parents were unsure if the word *anxiety* was the correct term to use, although they highlighted the children’s need for psychosocial support in the first phase. The adults were of the opinion that the word *anxiety* may be too strong a term to use when talking to young children and that they might find it challenging to understand the meaning of anxiety:

No, I don’t know. But anxiety is one of those adult words. “Oh, I have anxiety”. But it’s almost like a...anxiety, it should be classified as anxiety, but worry is something...I think children will recognize.

209; mother of a 12-year-old boy

##### Mapping the Journey to Facilitate Recall

The children liked features such as the schedule and the calendar that could help them remember what was planned, how the treatment worked, and how they felt each day. The schedule was regarded as a way of offering the children and parents a simple overview of the week:

You can see what’s going on and that the doctor can put in, yes, but around this time I’ll come in and talk to you, and around this time you’ll change the infusion (drip) or something.

101; girl, 14 years

The health care professionals were positive about the additional notes feature, as it was a way of getting to know the child and allowed them to tell their narrative. Meanwhile, parents saw it as a personal space for their child to make short notes that he or she could remember and write questions. They also saw the notes feature as a possibility for the child to document their treatment journey and write down things they were looking forward to.

The children proposed features that, in their opinion, were lacking in the design, such as being able to check off parts that had been completed on the schedule. The children also suggested the inclusion of information about their disease, common symptoms, and a treatment plan, so that they could understand themselves how their bodies are going to change and easily explain this to their friends:

Friends ask day in, day out, day in, day out. And it’s really hard because it’s so hard to explain.

110; girl, 7 years

##### Design of the Mock-up

The health care professionals and the parents wondered if the PicPecc app needed to be age adjusted; for example, if the pets and pictures were too childish for older children. Some parents thought it was too childish for adolescents, whereas others thought it looked good. The children themselves thought the design was simple and easy to understand but not childish:

I think it’s very nicely laid out and looks good etc. It’s not too difficult for five-year-olds and not too childish then for those who are older.

102; girl, 14 years

The health care professionals and parents felt that the design of the PicPecc app facilitated user-friendliness and appreciated the read-aloud feature.

The children wanted different ways of personalizing their avatars and pets, with both real and fantasy animals in the pet section. They also talked about being able to name and change different features of their pets.

All groups commented on the chosen symbols, as they found them difficult to interpret and thought that if pictures and picture schedules were used, they needed to be accurate. The children needed to see what was going to happen to feel safe. They also had difficulty in understanding some of the words used in the mock-up version of PicPecc; for example, care plan and estimates.

A part of the mock-up included creating a care plan. Children and parents found it difficult to understand the usefulness of this part. Children struggled with the word *estimates* and suggested that *estimates* be rewritten as *how do you feel?*

#### The PicPecc App

The mobile PicPecc app comprises a number of pages where children can describe how they are feeling using icons and a faces thermometer scale. On the home page, there are three options—record symptoms, access the gaming function (collecting animal icons that can be included in the child’s profile), and access an area where the data are displayed in the form of customizable statistics. A setting function also allows for customization of the sounds, spoken text, or notifications. The child is represented in the app by an avatar of their choice. Symptom location can be described on a body outline. There is a support and help page where the child can obtain information about their condition and tips and ideas about how to feel better. This can be linked to an external webpage.

## Discussion

### Principal Findings

There were 2 phases in this study. In the first phase, information from participants in stage 1 and experts from stage 2 formed a PicPecc mock-up in collaboration with UX specialists in stage 3. Both participants from stage 1 (phase 1) and new participants tested the mock-up in phase 2. The participants in phase 2 stated that the mock-up was accessible, affordable (in this case, the value of spending personal resources and time with the app), and acceptable. The results generally emphasized that the potential to support symptom management was a beneficial aspect of PicPecc and that children can find symptom relief within the app. Symptom relief is an important aspect of pediatric care and an essential part of the care process [61].

In stage 1, the children with long-term illness wanted to describe symptoms beyond pain, and parents and health care professionals confirmed the relevance of including anxiety, fatigue, fear, and nausea. This is consistent with previous work examining symptom distress in children with cancer [8]. The most frequently prioritized symptom assessed by the participants was anxiety, followed by fear. Similar findings have been highlighted in previous research [8,62]. PicPecc aims to support the child and the parents in discussing distressing issues and support the health care professionals in raising issues other than pain and nausea. PicPecc strives to facilitate communication and has the potential to be a tool that helps health care professionals listen to the child.

In stages 2 and 3 of phase 1, researchers cooperated with the participants to innovatively translate their needs into the PicPecc mock-up. Adaptations were made based on UD and person-centered care. The participants suggested that digitalization may facilitate the assessment of symptoms using a faces thermometer scale, which the participants described as useful in assessment, ranging from feeling good (green and smiley face) to worse (red, sad, and crying face). This type of traffic light system has previously been described to manage symptoms such as pain on a scale of 0 to 10 but is sometimes not enough to reflect the intensity level [63].

In stage 1 (phase 2), the children commented that the PicPecc mock-up was not too childish. This is in line with previous research identifying determinants that might have an impact on access to health care [64]. The design of the mock-up was accessible and acceptable to all children; it was simple and easy to use. UD might improve accessibility by adding sound, easy-to-read texts, and pictures that could facilitate communication across languages, cognitive developmental stages, or disabilities. This corresponds well with a study by Rodgers et al [22], stating that the method of assessing symptoms needs to be on a level that equals the child’s cognitive and developmental level. However, some of the parents and health care professionals in our study were concerned that the design was too childish. UD is a design that should not present an obstacle but rather use symbols and pictures to make the content more accessible for all people. Adults may see symbols and pictures as something for young children; however, as the results show, children of various ages can see the benefits of visualization.

In this study, a person-centered approach was adapted to pediatric care, which emphasizes the purpose of accessing the child’s stories [34]. The goal of such an approach is to empower the child to become more independent in their own care. Lin et al [1] stated a need for tools to enhance communication with health care professionals as children value empathic and respectful communication. Feelings of powerlessness and anxiety may arise when communication is perceived to be parent-centered or paternalistic [1]. PicPecc may have the potential to bridge the gap and open a dialog between the child and health care professionals. This dialog might help the child feel more independent, relieving the parents of the responsibility of being that bridge. Information and communication technologies can be a bridge between users who do not know the culture or language and the health care professionals [65]. The children in our study generally expressed a wish for autonomy and a possibility for communicating directly with the health care professionals about symptoms through mHealth tools.

In PicPecc, the story is told through the child’s own estimations and the note function, which can hopefully help the health care professionals recognize the child’s symptoms and focus on them. PicPecc intends to facilitate a supportive approach, which may enable more person-centered care. In addition, PicPecc is an attempt to create a digital tool that supports the child in expressing what they are feeling and provides information that may help them manage some of their symptoms. Symptom relief is a prerequisite for reducing long-term problems. For example, chronic pain in adulthood can progress from acute pain in childhood [26]. Thus, there is a need for a tool that helps children with symptom relief early and that is immediately available when a symptom occurs.

### Limitations

Six main limitations can be identified in this study. First, there is a risk of population selection bias, as the children and parents were recruited by the health care professionals who were treating them. Second, no child with congenital heart defects participated in phase 1 (stage 1). Third, the parents could have influenced the children’s answers, and fourth, it would have been valuable to have had a wider range of ages, as most of the participants were adolescents. Fifth, it is a limitation that the end users (ie, children, parents, and health care professionals) did not themselves participate in stages 2 and 3. Finally, the sixth limitation is that this is a description and mock-up of an app, and we have not yet been able to test the final version of PicPecc.

### Conclusions

The results from our study reveal a need for a tool that facilitates communication between children and health care professionals. Both parents and children stressed the importance of communication about feelings beyond symptoms of pain. With the potential to facilitate person-centered communication through UD, PicPecc is an advanced first attempt on how to provide support when dealing with multiple symptoms and conditions. PicPecc has the potential to open a dialog between the child and the health care professionals and addresses symptoms that may otherwise be overlooked. Over the past decade, the use of digitalization has expanded within health care. This study demonstrated the potential for using PicPecc as a digital support in clinical practice. Future phases should include usability testing and evaluation of the effects in hospitals and in home care settings.

The effectiveness of PicPecc to communicate symptoms and lead to symptom alleviation, thereby improving well-being and HrQoL, will be assessed in the next phase of the project. Representative populations of children in Sweden and South Africa will be identified through a randomization method and invited to participate in an evaluation study. In addition to PicPecc, standardized instruments for measuring symptoms and well-being will be administered to a group using PicPecc and a control group.

## Appendix

Multimedia Appendix 1 Parts of the mock-up.

Multimedia Appendix 2 Results of stage 1, phase 2.

## Abbreviations

HrQoL: health-related quality of life
mHealth: mobile health
PicPecc: Pictorial Support in Person-Centered Care for Children
UCD: user-centered design
UD: universal design
UX: user experience
