# Supplementary material for: The Development of an mHealth Tool for Children With Long-term Illness to Enable Person-Centered Communication: User-Centered Design Approach
Source: JMIR Pediatr Parent. 2022 Mar 8;5(1):e30364. doi: 10.2196/30364 (PMC8941441; doi:10.2196/30364)
Supplement: Multimedia Appendix 1 [file pediatrics_v5i1e30364_app1.docx]

Supplementary file 1. Parts of the mock-up

| **Universal Design** | **Parts of the mock-up** | **What was offered?** | **Design of the mock-up** |
| --- | --- | --- | --- |
| Layout | Home page  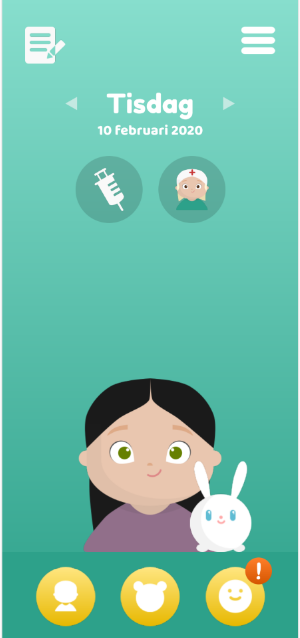 | Notes, todays date, a picture schedule, my page, assessments, reward system, statistics, information and settings. | A notepad in the upper left corner, a symbol in the upper right corner (settings, information, and statistics). The date in the middle together with a picture of a syringe and a nurse. Adina and a pet. At the bottom there are three yellow symbols for ‘my page’, ‘reward system’ and ‘assessments’. The background colour is green. |
| Adaptation | Sound | When you push the text, it will be read aloud. | All text can be read aloud. |
| Adaptation | Settings  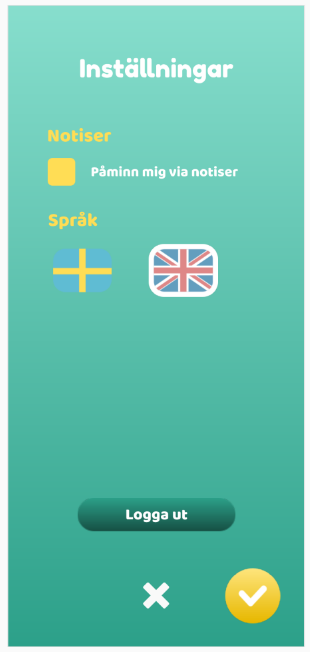 | Settings (notification, language (Swedish, English) and a log off function). | Settings in the top part of the page in white text. A Swedish and an English flag to represent the different languages you can choose from and a log-off function that contains pictorial support. |
| **PCC** |  |  |  |
| Personalized | Avatar  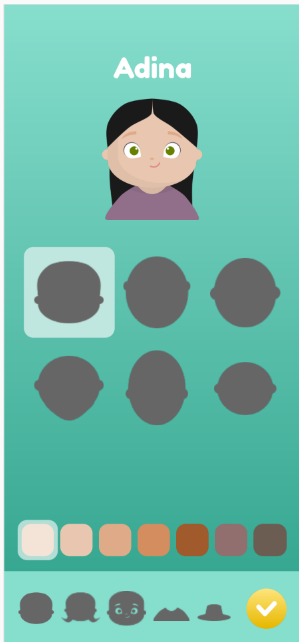 | You can choose the shape of the face, skin tone, haircut, and accessories like hats and sweaters. | A girl is presented with light skin, dark hair, and a purple sweater. The avatar is called Adina. |
| Personalized | Reward system  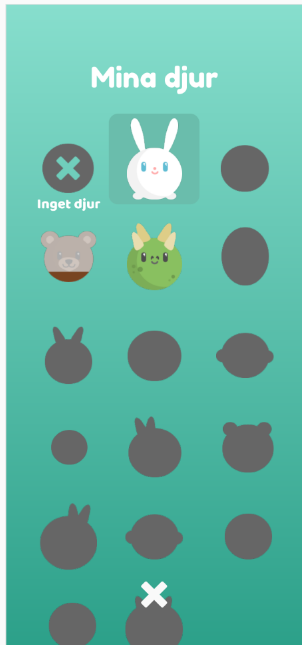 | When the child has assessed their symptoms, the pets will be unlocked. The child can choose which of the unlocked pets they want (or none) presented along with their avatar. | At the top, a white text that says ‘My pets’  after which is an x and the text ‘no animal’; next to it is a white bunny and then multiple shadows that symbolize other animals. One animal has started to be filled with colour. |
| Narrative | Assessments  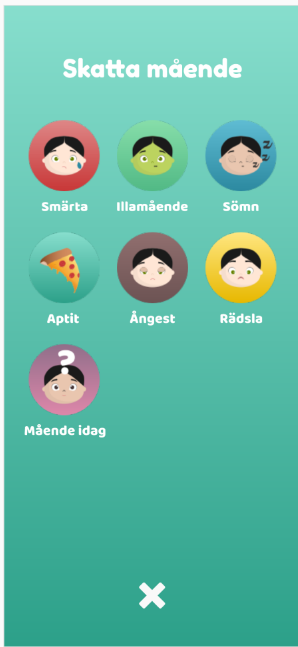  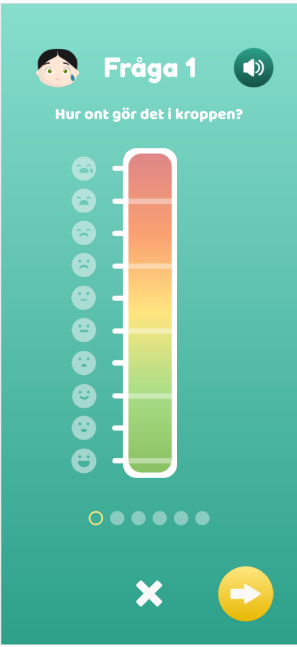  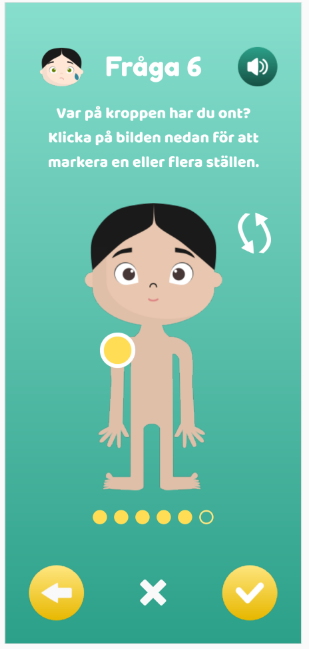 | Here the child can choose to assess anxiety, appetite, fatigue, fear, how you feel today, nausea and pain. When you press on ‘pain’ (the only one that shows how the system works) you will come to different questions about pain, pictures to make it easier (a thermometer scale, a body and then a summary of the questions you have answered). | At the top, a white text says, ‘Assess how you feel’. There are different pictures of faces, under which are descriptions of what each one represents (anxiety, appetite, fatigue, fear, how you feel today, nausea and pain). |
| Narrative | My page  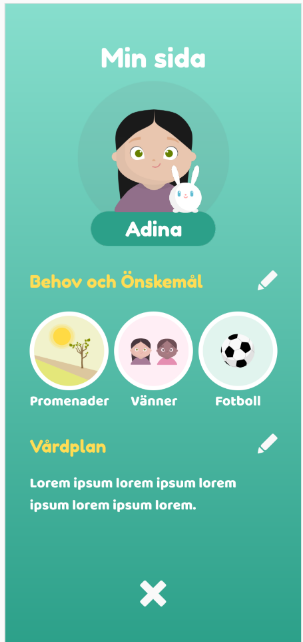  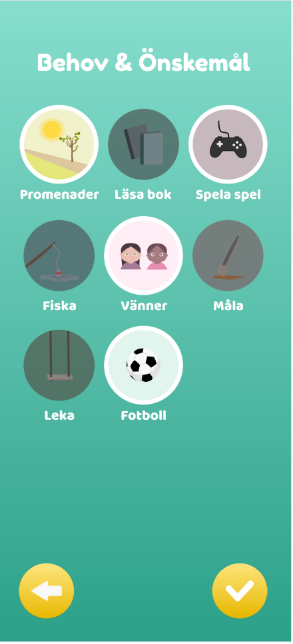 | The child can choose different pictures that symbolize what he/she likes and needs to feel happy. They can write a care plan and special requests they want the healthcare professionals to know. | The avatar and the pet can be seen in the upper part of the page along with the text ‘My Page’.  Then, in yellow text, ‘Needs and wishes’, with three pictures under the text showing walking, friends, and soccer. The last text is ‘Care plan’ in yellow. |
| Partnership | Coping strategies  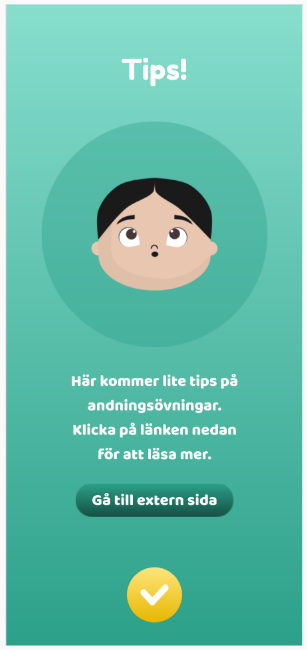 | You can press a button to go to an external site, e.g. for coping strategies. | A page that can be adapted to predetermined cut-off scores on the assessment scales. There is a link to an external page of coping strategies. |
| Documentation of partnership | Picture-based schedule  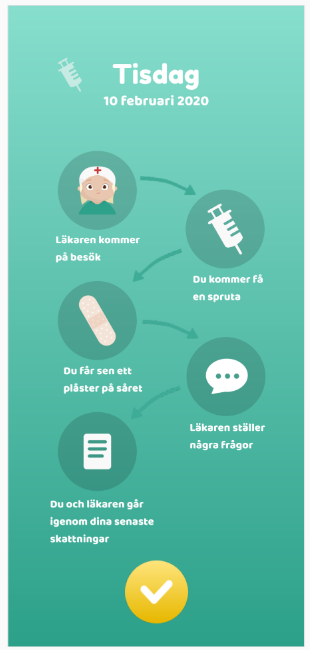 | Pictures show what will happen during the hospital visit that day. | The day and date are at the start. Pictures of a nurse, syringe, band-aid, a speech bubble, and notepad show the flow of the visit from top to bottom with arrows to guide the child in the right way. |
| Documentation | Notes  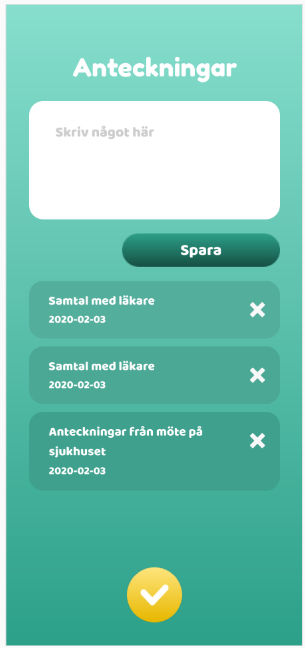 | Here the child can choose what they want to write. | At the top, ‘Notes’ in white text, followed by a white textbox where the child can write. A ‘save’ button and then different dark green textboxes with notes like ‘Dialogue with physician’ and the date. |
| Documentation | Statistics  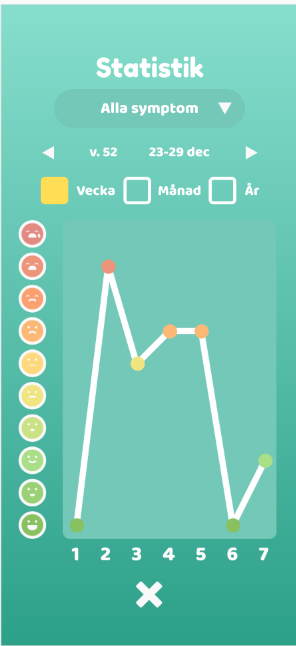 | Statistics of the child’s assessments are shown in a diagram where you can choose to display weeks, months, and years, all symptoms or one symptom at a time. | When you press the symbol, statistics will appear. At the top it says statistics in white, then you can choose a symptom or all, there are boxes that say day, week, year and at the bottom, there is a diagram that shows the assessments. |
