# Supplementary material for: The Development of an mHealth Tool for Children With Long-term Illness to Enable Person-Centered Communication: User-Centered Design Approach
Source: JMIR Pediatr Parent. 2022 Mar 8;5(1):e30364. doi: 10.2196/30364 (PMC8941441; doi:10.2196/30364)
Supplement: Multimedia Appendix 2 [file pediatrics_v5i1e30364_app2.docx]

| Supplementary file 2. Results of stage 1, phase 2 | | | | | |
| --- | --- | --- | --- | --- | --- |
| **PCC** | **Parts of the mock-up** | **Children** | **Parents** | **Health care professionals** | **Themes** |
| Personalized options | Choose an avatar  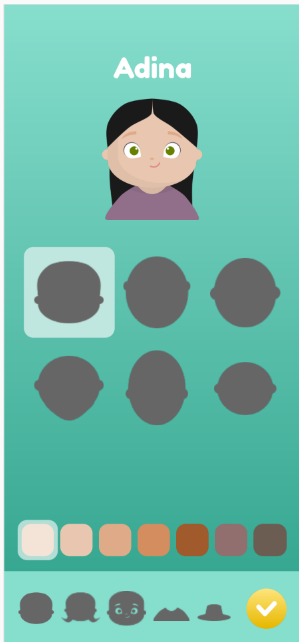 | I will create the avatar the same day we download the app. [106; girl, 9 years] | So that’s her then, as she normally looks. It should feel like hers. [210; mother of a 7-year-old girl] | It’s fun if you can create your own character. [316; focus group, nurse in paediatric cardiology]  You could make your own character and so on; it was great that you could create yourself. [308; female nurse] | Design of the mock-up |
| Personalized | Reward system  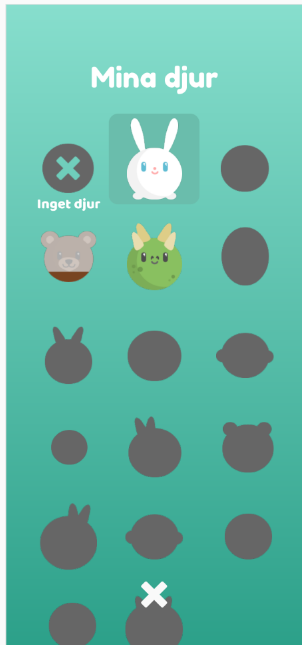 | Yes, and it’s very tempting to go in and do something when you have this pet as a point. [109; boy, 12 years]  I will probably fill it in every day to get an animal. [106; girl, 9 years] | But perhaps more open, so that it’s not just animals but “my interests”... because just animals, maybe it’s attractive for younger children. [201; mother of a 14-year-old girl] | Because they’re actually a bit childishly designed as well. Suitable for many who are younger that way. [307; focus group, nurse in a paediatric ward] | Design of the mock-up |
| Narrative | Assessments  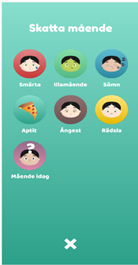  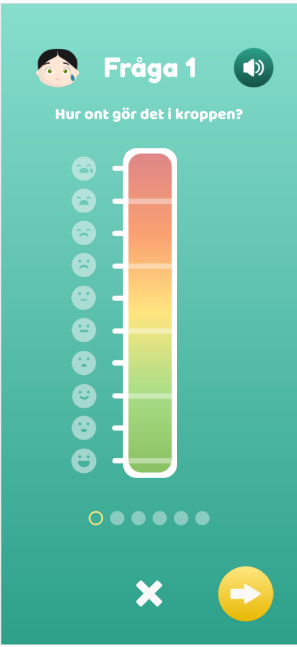  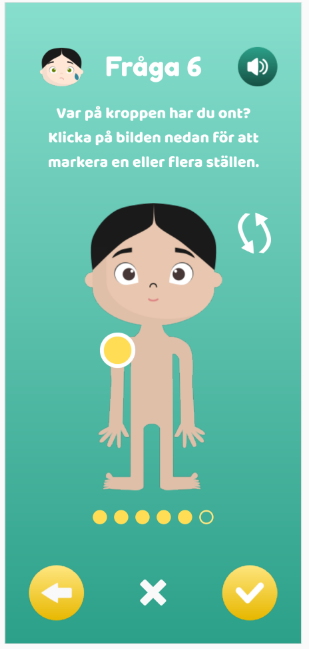 | I think it could have been nice to have “I don’t know what to do” in an app like this. “I have breathing problems, what should I do?” so there it is. [109; boy, 12 years]    It can show the nurses how much pain you have had. [106; girl, 9 years] | This could be a great tool for the staff here as well, a very good complement. Surely it’s much easier to get them to…interact with you. [202; mother of a 14-year-old girl]  Psychological health, it’s an area where I think a lot more can be done. [201; mother of a 14-year-old girl] | For nausea it can work, because it’s like, I feel a little sick or so. And fatigue too: a little tired or very tired. So appetite is perhaps the one that is most difficult then…fear and anxiety, they work too. [317; female nurse]    It could be used as a starting conversation during rounds. [307; focus group, nurse in a paediatric ward] | Need for an easy tool to assess symptoms and facilitate communication    Different perspectives on provided and perceived support  Design of the mock-up |
| Narrative | My page  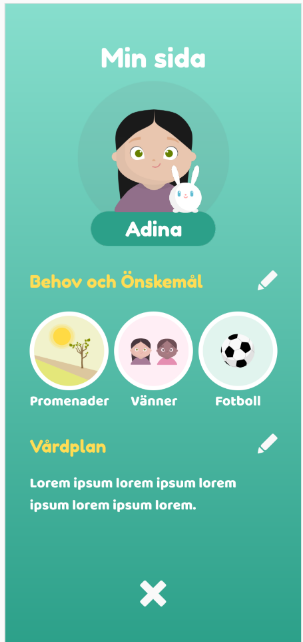  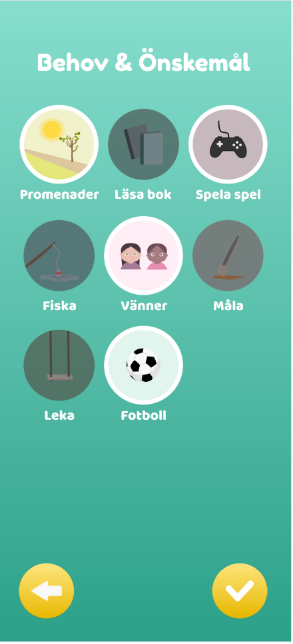 | What does it mean? Our plan? [110; girl, 7 years]  What you could do? Or…? [111; boy, 12 years]  I don’t know really. What you do when you’re not in hospital and stuff maybe. [105; girl, 8 years] | She has a diary where she explains what to do when they’re changing her…because it’s painful. [208; father of a 10-year-old girl] | What they like and how they want it. Also extremely important. [306; female physician] | Need for an easy tool to assess symptoms and facilitate communication |
| Documentation of the partnership | Picture-based schedule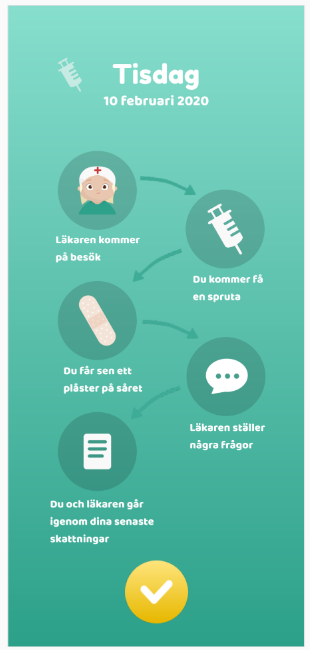 | I like it, like if you have a plan and you know what’s going to happen and so on. [102; girl, 14 years]  The most useful thing for me personally is the schedule. I think it’s great, so you can see what will happen… [101; girl, 14 years] | I can think that, for a newly diagnosed child just getting started, these steps could be very important. To know what will happen next. [210; mother of a 7-year-old girl]  It should be easy for them to understand because you’re not always that alert when you’re in pain, so it must be easy. And it is. [204; mother of a 16-year-old boy] | I think that’s great because then they’ve also gone through it thoroughly at home, so the children are prepared, and the parents, they have gone through it thoroughly at home, and then you can…I think that sounds great. [304; female nurse] | Different perspectives on provided and perceived support |
| Documentation | Notes  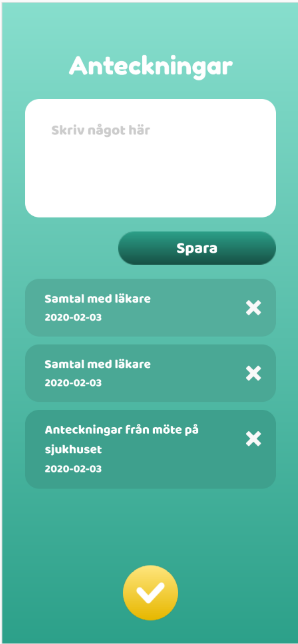 | It’s always good if you want to write something down, or if you have a question for the doctor. [101; girl, 14 years]  If you want to write, just for yourself, like a diary. [104; boy, 16 years]  I would write notes about how it went and how I felt after and before. [111; boy, 12 years] | Yes, it is meaningful because you can…when there’s a planned visit, you need to ask the question, you don’t want to forget. [202; mother of a 14-year-old girl]  Maybe it’s more interesting for parents. Or I don’t know, but children tend not to be so organised that they want to write… [201; mother of a 14-year-old girl] | I think that it is their own, actually. [305; female nurse]  Hmm, here is a place you can write notes so the children can, for example, write their goals for the visit…you give the child a space to think for themselves, express their wishes. [311; male nurse] | Mapping the journey to facilitate recall    Different perspectives on provided and perceived support |
| Documentation | Statistics  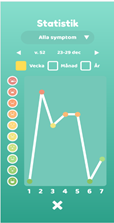 | You can see everything. If it gets better and better, or if it has become worse, you can see it. [102; girl, 14 years]  It feels like a great way, and that there is kind of a smiley up here in the corner, that “your average rating this week is yellow smiley”. “Oh, what have I done that makes me feel that way?” [109; boy, 12 years] | Nice to be able to follow too, you can see when everything is good so that you don’t forget those bits. Because you feel great sometimes. Right? [203; mother of a 14-year-old girl] | I think that the statistics is what helps us the most, to see how serious it is for the child. [315; male oncologist] | Mapping the journey to facilitate recall  Need for an easy tool to assess symptoms and facilitate communication |
